# Persistent Features of Laryngeal Injury Following Endotracheal Intubation: A Systematic Review

**DOI:** 10.1007/s00455-023-10559-0

**Published:** 2023-02-11

**Authors:** Eileen Kelly, Julia Hirschwald, Julie Clemens, Julie Regan

**Affiliations:** 1 Department of Clinical Speech & Language Studies, Trinity College Dublin, Dublin, Ireland; 2 Highly Specialist Speech & Language Therapist, Adult Critical Care Unit, Royal London Hospital, London, UK; 3 Patient and Public Representative, London, UK

**Keywords:** Intubation, Post-extubation, Laryngeal function, Laryngeal injury

## Abstract

This systematic review examined (i) prevalence, severity, and impact of persistent post-extubation laryngeal injury beyond hospital discharge and (ii) differences in persistent laryngeal injury between COVID-19 and non-COVID-19 populations. The review was completed following PRISMA-2020 guidelines. Four databases (PubMed, CINHAL complete, EMBASE, Web of Science) were searched (inception to March 2021). Screening, full text review and data extraction were completed by two reviewers. Primary outcomes were swallow, voice and cough and airway measures obtained after hospital discharge. Quality assessment was measured using Downs & Black Tool and Johanna Briggs Institute Checklist for Cohort Studies. Meta-analysis was not completed due to study heterogeneity. Six cohort studies were included. Total number of participants across the included studies was 436. ICU admission diagnoses included respiratory disease 46% (COVID-19 and non-COVID-19), sepsis 14%, non-sepsis-related organ dysfunction 9%, general medical 11%, general surgical 10%, trauma 2%, ENT 0.6% and other not specified by authors 7%. Outcomes were obtained between 2 and 60 months post hospital discharge. Assessment methods included endoscopic evaluation, clinician ratings and patient-reported outcomes. Persistent features of laryngeal injury identified were airway abnormalities (18.9–27%), dysphonia (13.2–60%) and dysphagia (23–33%). Persistent laryngeal injury was associated with ICU length of stay, respiratory diagnosis and tracheostomy. Study quality ranged from poor-good. This is the first systematic review to examine post-extubation laryngeal injury beyond hospital discharge. Significant gaps in the literature were identified. Given the impact on clinical and patient outcomes, large scale, well-designed research is needed to guide post-ICU service delivery.

**Supplementary Information:**

The online version contains supplementary material available at 10.1007/s00455-023-10559-0.

## Introduction

Endotracheal tube (ETT) intubation is an essential component of intensive care management of severe respiratory disease, though the iatrogenic effects of intubation have potential for acute and chronic complications [1]. The ETT sits in a vulnerable anatomical region for laryngeal function, and as a result, injury to the larynx is a common occurrence, with an estimated prevalence of 83% immediately following extubation [1]. While many of these injuries are self-limiting, more severe injuries may persist and require intervention [2].

### Laryngeal Injury

Globally, 13–20 million patients in Intensive Care Units (ICUs) are intubated, with only a small fraction of patients emerging from intubation injury free [1]. Critically ill patients may transient or prolonged intubation, and many have conditions that predispose them to laryngeal injury [3]. Laryngeal injury may manifest as dysphagia, dysphonia, vocal cord paralysis, laryngeal oedema, granuloma and airway stenosis, though this is not an exhaustive list [1, 2]. Previous systematic reviews have investigated laryngeal injury in inpatient cohorts and reported incidence rates of airway, voice and swallow impairment as 13–31%, 76% and 3–62%, respectively [4–6].

### Long-Term Follow-Up

Despite a growing body of evidence within inpatient populations, research examining the prevalence of persistent laryngeal injury post-hospital discharge is lacking. The impact of laryngeal injury on functional recovery from critical illness represents a research gap [4–7]. Identification and management of these symptoms in the post-acute phase is clinically important, reducing complications for patients during a vulnerable time where medical setbacks may be detrimental to recovery [8–10].

### Coronavirus Disease-19 (COVID-19)

Severe Acute Respiratory Syndrome Coronavirus-2 (SARS-CoV-2) resulted in a worldwide COVID-19 pandemic in March 2020. [11]. For those where COVID-19 led to severe respiratory disease, invasive ventilation via ETT was required [12]. A distinctive characteristic of those intubated as a consequence of COVID-19 was the duration of ventilator reliance, up to twenty days and beyond [13]. Duration of intubation has previously been associated with dysphagia in adults with acute lung injury [4]. Prone ventilation was used widely in the COVID-19 cohort, with patients reported to have been in prone position for up to 17 h [14]. Though limited research exists to date, Regan et al. found prone ventilation amongst factors associated with post extubation oral intake status [15].

### Study Aims

(i) To systematically review the literature to examine the nature, severity, prevalence, and factors associated with features of laryngeal injury persisting beyond acute hospital discharge, in patients who underwent ETT intubation during ICU admission.
(ii) To examine differences in outcomes between COVID-19 and non-COVID-19 populations.

## Methods

### Protocol and Registration

The Preferred Reporting Items for Systematic Reviews and Meta-Analyses (PRISMA) 2020 statement was followed [16]. The study was registered on Prospective Register of Systematic Reviews (PROSPERO) (registration number: CRD42020223289). Due to the COVID-19 pandemic, the registration record was automatically published and eligibility was not checked by PROSPERO prior to publication.

### Information Sources and Search Strategy

Search strategy was created alongside a subject Librarian. Four databases were searched for peer-reviewed articles: PubMed, EMBASE, CINAHL and Web of Science, from inception to March 2021. Grey literature and/or preprints were excluded. Searches were completed in the English language only. The search strategy is provided in *supplementary appendix A.* Following the electronic search, citations were imported to the online platform Covidence [17].

### Study Selection

Title and abstract screening, full text review and extraction were completed independently by two authors. Disputes were resolved by a third-party reviewer. Given the limited research in this area, all study designs were considered for inclusion. Expert opinion, letters to the editor, commentaries and editorials were excluded. Data were then extracted to Microsoft Excel for results synthesis.

### Eligibility Criteria

Studies that met the following inclusion criteria were eligible: (1) adult participants ≥ 18 years old who underwent ETT intubation during ICU admission, (2) outcomes evaluated beyond acute hospital discharge. ICU is defined as full spectrum monitoring and life support for critically ill patients [18]. Persistent laryngeal injury was defined as injuries remaining beyond hospital discharge. This definition was selected rather than a specific timepoint, so as not to exclude patients who required longer admissions. Participants who had pre-existing or co-occurring conditions known to cause dysphagia (i.e. neurological conditions, head and neck cancer) were excluded.

### Outcomes

A list and definition for all outcomes were prepared in line with the PRIMSA 2020 statement [16]. Outcomes were selected with a co-author on this study who was a patient and public representative.

The primary outcomes for examination were as follows:

1. Instrumental voice measures (e.g. endoscopic measures of the larynx).
2. Instrumental measures of swallowing (e.g. fiberoptic endoscopic evaluation of swallowing (FEES)).
3. Instrumental airway evaluation (e.g. nasendoscopy).

Secondary measures were as follows:

1. Perceptual or clinically derived measures (e.g. grade, roughness, breathiness, asthenia and strain (GRBAS); [19]).
2. Patient-reported outcome measures (e.g. Voice Handicap Index-10 (VHI-10) [20]).
3. Functional measures (e.g. Functional Oral Intake Scale (FOIS) [21]).

### Data Extraction and Quality Assessment

Following full text review and consensus on articles for inclusion, articles were extracted from Covidence [17] to Microsoft Excel for analysis. One author completed data collection from the included full texts and inputted the data to Microsoft Excel.

### Data Synthesis and Analysis

Primary and secondary outcomes were collected, and analysis was carried out using descriptive statistics. General characteristics of included studies, baseline characteristics, patient demographics, assessment methods of included studies and factors associated with laryngeal injury were collated. COVID-19 studies were analysed as per the secondary aim.

### Assessment of Methodological Quality

Methodological quality was assessed by two authors using the Downs and Black checklist [22] and the Joanna Briggs Institute (JBI) Checklist for Cohort Studies Critical Appraisal Tool [23]. The Downs and Black Checklist was modified for the purpose of this review, as it has been successfully adapted in previous systematic reviews [24]. Criteria regarding interventions were omitted as they were not relevant to the aims of this study. The following ranges were used: excellent (18/20), good (13/20), fair (8//20) and poor (≤ 7). The JBI does not provide a range of scores that indicate the overall quality; however, higher scores are reported to represent better methodological quality [23].

## Results

### Study Selection

**Fig. 1:**
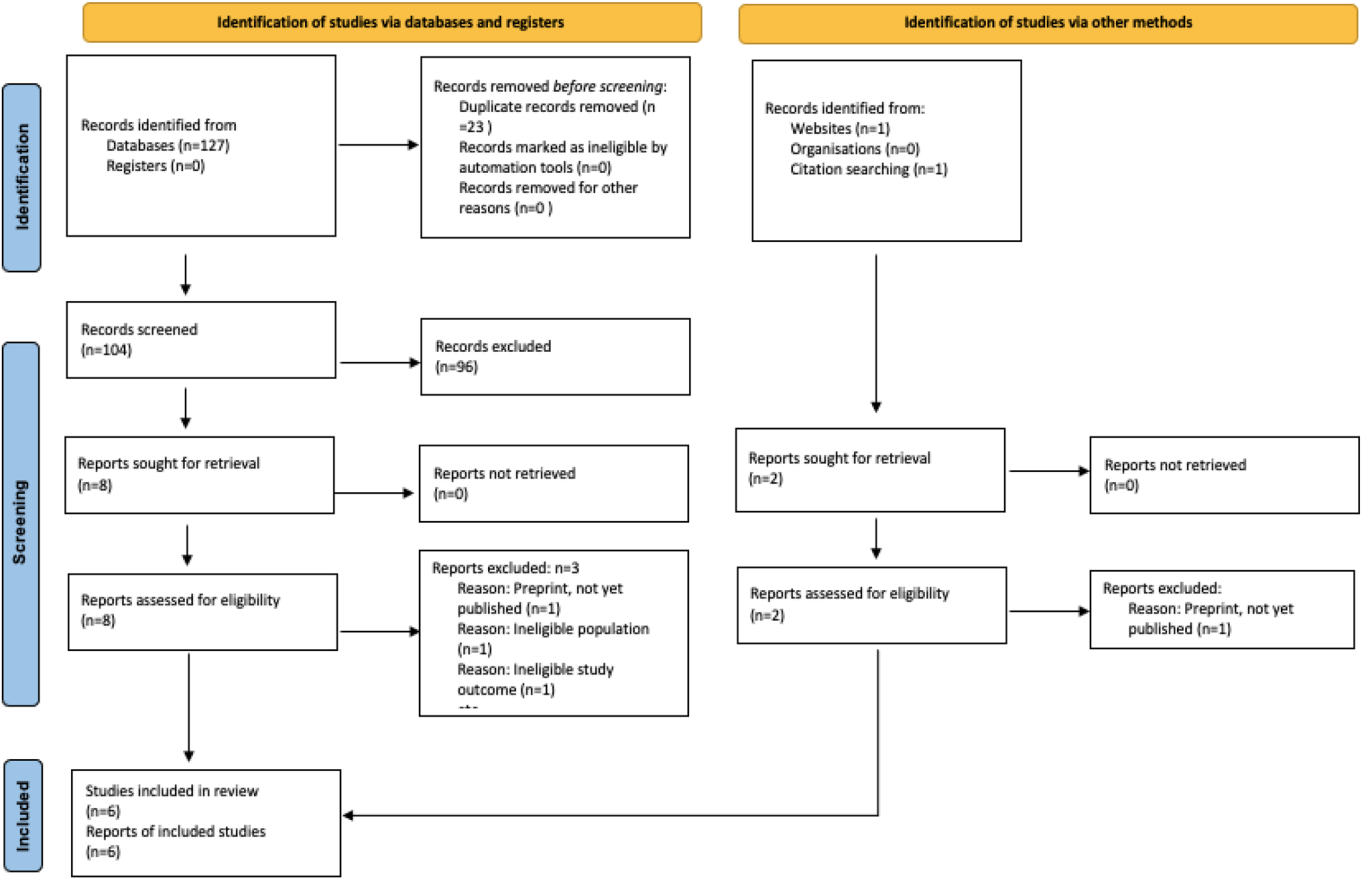
PRISMA diagram

Electronic search was completed on the 1st of March 2021. In total, 127 records were identified. Following extraction, 23 duplicates were removed, and 104 records were screened. 8 studies were assessed for eligibility during full text review, with six studies eligible for inclusion. Reasons for exclusion are outlined in the PRISMA [16] diagram shown in Fig. 1.

### Characteristics of Included Studies

All included studies were prospective cohort studies and were published in English. The total number of participants across the included studies was 436. The mean number of participants in each study was 73 (range 20–115). All studies reported higher percentage of male participants (mean = 65%). The mean age reported was 59 years (range 33–77 years).

Five of the included studies included ICU admission diagnoses (*n* = 336 participants). ICU admission diagnoses included 46% respiratory disease including COVID-19 (total *n* = 153: non-COVID-19 *n* = 92, COVID-19 *n* = 61), 14% sepsis (*n* = 48), 9% non-sepsis-related organ dysfunction (*n* = 30), 11% general medical diagnoses (*n* = 38), 10% general surgery diagnoses (*n* = 34), 2% trauma (*n* = 6), 0.6% ear nose and throat diagnosis (*n* = 2) and 7% other (not specified by author [27] (*n* = 25). Two of the six included studies reported outcomes on COVID-19 cohorts [25, 26]. No study provided the indication for intubation via ETT, grade of intubation, indication for tracheostomy or method of tracheostomy insertion. 26% of total participants were reported to have a tracheostomy during their inpatient phase (*n* = 116). Beyond hospital discharge, 1% of participants had a tracheostomy (*n* = 5).

Duration of intubation was reported by all included studies, with a mean duration of 12 days. Longer durations of intubation (mean = 30 days) were reported in the COVID-19 studies [25, 26]. When these studies were excluded, the mean duration of intubation reduced to 6 days. Duration of ICU admission was reported by three studies, with a mean duration of 12 days (range 4–22 days) [27–29]. Hospital length of stay was reported by one study [27]. Table 1 outlines characteristics of included studies.

**Table 1:** Characteristics of included studies

Study, *n* = sample size	Brodsky et al. [27], *n* = 115	Zielske et al. [28], *n* = 60	Nixon et al. [29], *n* = 100	Shinn et al. [3], *n* = 100	Naunheim et al. [25], *n* = 20	Rouhani et al. [26], *n* = 41
Study design	Prospective cohort study	Prospective cohort study	Prospective cohort study	Prospective cohort study	Prospective cohort study	Prospective cohort study
Age: mean, (range)	48 (40–57)	68 (NR)	60 (NR)	59 (43–67)	59 (33–77)	57 (32–77)
Sex (% male)	52%	70%	61%	62%	75%	68%
Baseline health Status						
• Charleson Comorbidity Index	1	NR	NR	3	NR	NR
• APACHE II score (mean)	23	21	16	NR	NR	NR
ICU admission diagnosis	Respiratory (*n* = 66), non-pulmonary sepsis (*n* = 18), trauma (*n* = 6), other (*n* = 25)	Sepsis (*n* = 30), non-sepsis organ dysfunction (*n* = 30)	General medical (*n* = 38), surgical (*n* = 34), respiratory (*n* = 26), ENT primary diagnosis (*n* = 2)	NR	COVID-19 (*n* = 25)	COVID-19 (*n* = 41)
Reason for intubation	NR	NR	NR	NR	NR	NR
Grade of intubation	NR	NR	NR	NR	NR	NR
Duration of intubation, days (range)	7 (5–11)	9 (1–16)	8 (4–8)	3 (NR)	22 (9–33)	24 (10–40)
ETT size, mean (range)	NR	NR	NR	7.5 (7.0–8)	7.5 (3.5–8)	NR
ETT cuff pressure	NR	NR	NR	< 30 mmHg	NR	NR
Tracheostomy, Y/N (number of patients)	N	Y, *n* = 18	Y, *n* = 48	NR	Y, *n* = 9	Y, *n* = 41
Duration with tracheostomy, days (range)	N/A	NR	NR	NR	16 (0–27)	15
ICU length of stay, days (range)	11 (8–15)	22 (0–34)	4	NR	NR	NR

*N/A*, not applicable, *NR* not reported

### Nature, Prevalence and Severity of Laryngeal Injury

Assessments used to evaluate airway, voice and swallow outcomes beyond hospital discharge included instrumental evaluation of the larynx [25, 26, 28], clinical evaluation [26, 28, 29] and patient-reported outcomes [3, 26, 27]. Outcomes were evaluated across a range of timepoints. Assessment beyond hospital discharge took place between 8 and 16 weeks in most studies [3, 25, 26, 28], with one study performing multiple follow-up assessments up to 240 weeks post hospital discharge [27].

Data were provided on *n* = 386 participants beyond hospital discharge. Of the included studies, four reported swallow outcomes beyond hospital discharge [25–28] and four reported voice outcomes [3, 25, 26, 29]. Two studies reported on airway, swallow and voice outcomes beyond hospital discharge [25, 26].

#### Airway Outcomes

Airway outcomes were evaluated using laryngoscopy [25], stroboscopy [25] and flexible nasendoscopy [26]. Both studies examined COVID-19 cohorts with a total of *n* = 61 participants. The prevalence of airway abnormalities reported across both studies was 18.9–27%, with unilateral vocal fold palsy most reported (7.9–40%). Other laryngeal pathologies reported included glottic stenosis (15%), subglottic stenosis (5.3–10%) and granulation tissue (10%). Severity of airway injury was not reported.

#### Voice Outcomes

Dysphonia was reported by four studies, with prevalence of 13.2–60% reported [3, 25, 26, 29]. Two studies used laryngoscopy [25] and one study used stroboscopy [26]. Clinician-reported outcomes were included by two studies [26, 29]. Two studies provided patient-reported outcomes [3, 26]. There was a discrepancy between clinician and patient-reported outcomes when rating the prevalence of dysphonia [26]. Severity was reported by one study [29], with 16% of patients reported as severely dysphonic and 33% moderately dysphonic.

#### Swallow Outcomes

Dysphagia beyond hospital discharge was reported by four studies [25–28]. Total number of participants evaluated was *n* = 236. Prevalence was reported as 23–33% [25–28]. One study used instrumental swallow evaluation (FEES) to report dysphagia outcomes [28], using the Penetration–Aspiration Scale (PAS) [30] to grade the severity of aspiration. The same study reported severity as 16% and oral diet restriction as 21% [28]. Patient-reported outcomes were used by two studies [25, 26].

### Patient-Reported Outcome Measures

Three of the six studies included patient-reported outcome measures (PROM) [3, 26, 27]. One study [26] the VHI-10 [20] with 13.2% of patients reporting their voice as abnormal. 12.8% of patients reported symptoms of laryngopharyngeal reflux using the Reflux Symptom Index (RSI) [31]. 58% reported dysphagia on the Dysphagia Handicap Index (DHI) [32]. 30% of patients rated themselves as having an abnormal swallow on the Eating Assessment Tool-10 (EAT-10), [33]. 23% reported dysphagia using the Sydney Swallow Questionnaire (SSQ) [34].

### Factors Associated with Laryngeal Injury

Three studies performed multivariable regression model analysis to determine the factors associated with prevalence of voice and swallow outcomes [27–29]. Respiratory diagnosis on admission to ICU [29], duration of ICU admission [27] and the presence of tracheostomy [28] are reported as factors associated with dysphonia [29] and dysphagia [27, 28]. Factors associated with airway injury were not reported. Table 2 summarises the assessment methods, prevalence and severity of laryngeal injury.

**Table 2:** Nature, prevalence and severity of persistent laryngeal injury

Study, *n* = sample size beyond hospital discharge	Brodsky et al. [27], *n* = 115	Zielske et al. [28], *n* = 60	Nixon et al. [29], *n* = 83	Shinn et al. [3], *n* = 67	Naunheim et al. [25], *n* = 20	Rouhani et al. [26], *n* = 41
Outcome reported	Swallow	Swallow	Voice	Voice	Airway, voice, swallow	Airway, voice, swallow
Timepoint beyond hospital discharge, weeks	Multiple timepoints (12–240)	16	8	10	NR	8
*Method of assessment*						
• Instrumental		FEES			Laryngoscopy Stroboscopy	Nasendoscopy
• Clinical		FOIS [21]	VoiSS [35]			GRBAS [19] FOIS [21] WST [36]
• Patient-reported Outcome	SSQ [34]			VHI-10 [20]		VHI-10 [20] RSI [31] EAT-10 [33] DHI [32]
Prevalence of injury	Dysphagia: 23%	Dysphagia: 23%	Dysphonia: 49%	NR	Airway injury: 27%	Airway injury: 18.9%
					Dysphagia: 30%	Dysphagia: 30%
					Dysphonia: 60%	Dysphonia: 13.2–53.7%
Severity	NR	16% severe dysphagia	16% severe, 33% moderate	NR	NR	NR

*SSQ* Sydney Swallow Questionnaire [34], *FEES* Fibreoptic Endoscopic Evaluation of Swallow, *FOIS* Functional Oral Intake Scale [21], *VoiSS* Voice Symptom Scale [35], *VHI-10* Voice Handicap Index-10 [20], *GRBAS* Grade, Roughness, Breathiness, Asthenia, Strain [19], *WST* Water Swallow Test [36], *EAT-10* Eating Assessment Tool-10 [33], *DHI* Dysphagia Handicap Index [32], *NR* Not reported

### COVID-19 Patients

Two studies provided outcomes beyond hospital discharge on COVID-19 patients [25, 26]. Mean age of patients was 58 years. Age was wider ranging than non-COVID-19 cohorts (33–77 years). The mean duration of intubation reported for this cohort was 23 days, higher than the mean 6 days reported in the non-COVID-19 patients [3, 27–39]. One study reported that 100% of patients who underwent prone ventilation demonstrated laryngeal injury [25]. Airway abnormalities were reported as 27% [25] and 18.9% [26]. These included unilateral vocal fold immobility (40%), posterior glottic stenosis (15%), subglottic stenosis (10%), granulation tissue or edema (10%), laryngopharyngeal reflux (10%), posterior glottic diastasis (10%), muscle tension dysphonia (5%) and unrelated pre-existing conditions (15%) [25]. Rouhani et al. [26] reported unilateral vocal cord palsy in 7.9% and subglottic stenosis in 5.3%. Rouhani et al. [26] used clinical and patient-reported outcome measures to report on dysphonia and dysphagia rates. Reports of dysphonia varied from 13.2 to 53.7% [26]. Dysphagia was reported at 30% [25]. Naunheim et al. [26] reported both dysphagia and dysphonia in their outcomes but did not provide assessment methods.

### Quality Assessment

Quality assessment of included studies was undertaken by two authors. The JBI Checklist [23] and the Downs and Black Checklist [22] were completed on all included studies [23]. Items responsible for lower ratings included lack of reporting of confounding factors, outcomes not measured in a valid way and insufficient duration of follow-up period. The quality of the included studies ranged from poor to good [22]. Table 3 outlines the quality assessment completed.

**Table 3:** Quality assessment

Study	Downs & Black Checklist [22]		Quality Rating	Joanna Briggs Institute Checklist [23]	
	*Reviewer 1*	*Reviewer 2*		*Reviewer 1*	*Reviewer 2*
Brodsky et al., 2017	15/20	11/20	Fair-good	9/11	2/11
Nixon et al., 2010	15/20	8/20	Fair-good	8/11	1/11
Shinn et al., 2019	13/20	11/20	Fair-good	6/11	4/11
Zielske et al., 2014	17/20	11/20	Fair-good	10/11	5/11
Naunheim et al., 2020	8/20	2/20	Poor-fair	2/11	1/11
Rouhani et al., 2021	12/20	8/20	Fair-good	6/11	1/11

Key: Downs and Black quality assessment rating: excellent (18/20), good (13/20), fair (8//20) poor (≤ 7)

## Discussion

### Main Findings

To the authors’ knowledge, this is the first systematic review examining persistent features of laryngeal injury amongst adults who underwent endotracheal intubation, beyond hospital discharge. The prevalence of features of laryngeal injury was as follows; airway abnormalities 18.9–27%, dysphonia 13.2–60% and dysphagia 23–33% [3, 25–29].

Previous systematic reviews have investigated laryngeal injury in the acute phase [4–7]. While direct comparisons cannot be drawn due to the discrepancy in timepoints of assessment, previous research has reported the prevalence of laryngeal injury as 83% [4] dysphagia 41% [5] and dysphonia 73% [4]. Persistent laryngeal injury may be indicative of a more persistent impairment [2].

The cohorts across the included studies were heterogenous [25–30]. Of the studies who reported factors associated with laryngeal injury, respiratory diagnosis on admission to ICU [29], duration of ICU admission [27] and presence of tracheostomy [28] were significantly associated with dysphonia [29] and dysphagia [27, 28]. Factors associated with airway injury were not reported. This is inconsistent with previous research, which has identified the ETT size and cuff pressure [37], duration of intubation [4] and high cuff pressure impacting on the recurrent laryngeal nerve [8]. Variability in the data provided across all studies precluded statistical analysis of factors associated with persistent laryngeal injury. Future research should focus on establishing a core set of included characteristics, which would strengthen statistical analysis.

Prone ventilation was reported by the two COVID-19 studies [25, 26], though statistical analysis on the significance of this variable was not reported. Prolonged duration of mechanical ventilation has demonstrated up to 30% to muscle mass loss, and in combination with prone ventilation this may be expedited [43]. Mean duration of intubation reported in the COVID-19 cohorts was notably higher at 23 days [25, 26]. A combination of these factors may contribute to sarcopenia-related dysphagia, which has been demonstrated in elderly patients [38]. The impact of prone ventilation on laryngeal function warrants further research.

### Clinical Implications

Variability in assessment methods, outcomes and timepoints of assessment reported in this systematic review add to an incomplete understanding of the long-term effects of laryngeal injury, as has been highlighted in previous research [9]. ICU represents a heterogenous cohort, and consistent reporting of patient characteristics and factors that impact laryngeal function (such as duration of intubation) are required. Variation in assessment methods and functional outcome data limit interpretation of findings. A holistic assessment of the ICU patient beyond hospital discharge, which may encompass instrumental and patient/clinician-reported outcomes is needed to develop the evidence base. Assessment across multiple timepoints will provide greater insight to the trajectory of these persistent injuries. A patient and public representative co-author on this study bring the experience of undergoing a prolonged ICU admission. They highlighted the importance of quality of life-related measures in the assessment toolkit. Additionally, the author emphasised the need to consider the patients cognitive and emotional recovery post-ICU during follow-up assessment.

### Features of Laryngeal Injury-A Lasting Legacy of Critical Illness?

Only recently has attention turned to the impact of intubation on the larynx for long-term survivors of ICU admission [39]. Notably, only one study followed up patients over multiple timepoints [27]. No study reported that assessment took place as part of multidisciplinary team follow-up clinic or provided further information on the services required by the included participants. It is unlikely that persistent features of laryngeal injury exist in isolation from other post intensive care symptoms, given that more than half of all ICU patients present with new disability at 6 months post hospital discharge [40]. Understanding the trajectory of recovery and follow-up services required may further guide discussions regarding ICU follow-up and aid prognostication for this complex patient cohort.

### Limitations

The authors acknowledge several limitations in this study. Searches were conducted in the English language only. No grey literature or hand searches were completed. Data needed to address the research questions were missing from the included studies. The reported prevalance and severity of the persistence of dysphagia and dysphonia in this study is largely based on patient-reported outcomes. The paucity of instrumental assessment to evaluate voice and swallow outcomes means the findings may not give a true representation of the level of impairment. The small sample size of included studies precluded statistical analysis and the heterogeneity of the studies meant meta-analysis was not possible.

### Future Direction

A shift in focus towards examining the lasting effects of critical illness and improving survivor outcomes is required to fully address the long-term consequences of intensive care [41, 42]. Unfortunately, the current available evidence on airway, swallow and voice outcomes does little to address these gaps. The lack of consistent assessment methods and outcome measurement prohibits conclusions on the nature, severity and prevalence of these injuries. The need for well-designed, larger scale research is needed to provide more definitive analysis persistent features of laryngeal injury. Patient and public involvement at conception of the research design will inform the outcome measurements needed for this heterogenous population.

## Conclusions

Limited evidence exists on persisting features of laryngeal injury beyond hospital discharge. This is the first systematic review to address the nature, severity and prevalence of persistent features of laryngeal injury in those who underwent endotracheal intubation during ICU admission. Significant gaps in the existing literature were identified. Persistent features of laryngeal injury reported at hospital discharge were as follows: airway abnormalities 18.9–27%, dysphonia 13.2–60% and dysphagia 23–33%. Well-designed, larger scale research is needed to determine the most appropriate assessment and management of these injuries in the post-acute phase.

## Supplementary Information

Below is the link to the electronic supplementary material.

Supplementary file1 (DOCX 30 KB)
