# Supplementary material for: Persistent Features of Laryngeal Injury Following Endotracheal Intubation: A Systematic Review
Source: Dysphagia. 2023 Feb 11;38(5):1333–41. doi: 10.1007/s00455-023-10559-0 (PMC9922098; doi:10.1007/s00455-023-10559-0)
Supplement: Supplementary file 1 — Supplementary file1 (DOCX 30 KB) [file 455_2023_10559_MOESM1_ESM.docx]

**Appendix: Supplementary Evidence**

**Appendix A**

Electronic Search Strategy

| (“Critical Illness”[Mesh]) OR (“Critical Care”[Mesh]) OR (“Intensive Care Units”[Mesh]) OR (intensive care unit*[Title/Abstract] OR critical care [Title/Abstract] OR critical illness[Title/Abstract] AND (intratracheal intubation[Title/Abstract] OR (“Intubation, Intratracheal”[Mesh]) AND (“Deglutition Disorders”[Mesh]) OR (deglutition[Title/Abstract] OR swallow* [Title/Abstract] OR laryn* [Title/Abstract]) AND (randomised controlled trial[pt] OR controlled clinical trial [pt] OR randomized[tiab] OR randomised [tiab] OR randomly[tiab] OR trial [tiab] OR placebo [tiab] OR drug therapy [sh] OR groups [tiab] OR (follow-up) [tiab] OR retrospective {tiab} OR prospective[tiab] AND (study[tiab]) OR studies[tiab]) NOT (animals[mh] NOT humans[mh]) |
| --- |
